# Supplementary material for: Synthesis and Properties of Ethylene Imine-Based Porous Polymer Nanocomposites with Metal Oxide Nanoparticles
Source: Molecules. 2025 Aug 31;30(17):3574. doi: 10.3390/molecules30173574 (PMC12430365; doi:10.3390/molecules30173574)
Supplement: Supplementary file 1 [file molecules-30-03574-s001.zip › molecules-3818347-supplementary.pdf]

Supporting Information

Synthesis and Properties of Ethylene Imine-Based Porous Polymer Nanocomposites with Metal Oxide Nanoparticles

Naofumi Naga [1,2,*], Julia Janas [3], Tomoya Takenouchi [2] and Tamaki Nakano [4,5]

1 College of Engineering, Department of Applied Chemistry, Shibaura Institute of Technology,
Toyosu Campus, 3-7-5 Toyosu, Koto-ku, Tokyo 135-8548, Japan
2 Graduate School of Science & Engineering, Shibaura Institute of Technology, Toyosu Canpus, 3-7-5 Toyosu, Koto-ku, Tokyo 135-8548, Japan; ad20079@shibaura-it.ac.jp
3 MSc In Advanced Nano and Bio Materials MONABIPHOT, Wrocław University of Science and Technology, Wybrzeże Stanisława Wyspiańskiego 27, 50-370 Wrocław, Poland; janas.julia@gmail.com
4 Institute for Catalysis, Hokkaido University, N 21, W 10, Kita-ku, Sapporo 001-0021, Japan; tamaki.nakano@cat.hokudai.ac.jp
5 Integrated Research Consortium on Chemical Sciences, Institute for Catalysis, Hokkaido University, N 21, W 10, Kita-ku, Sapporo 001-0021, Japan
* Correspondence: nnaga@sic.shibaura-it.ac.jp

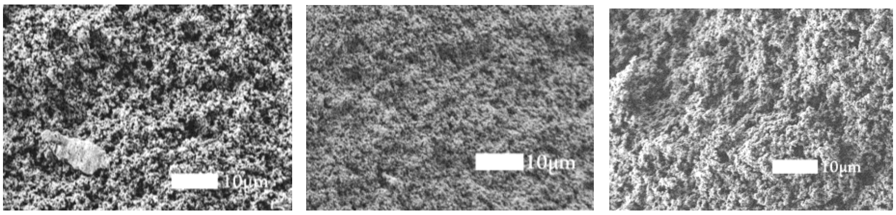

ST-20, 2.0 wt%, 20 °C ST-20, 4.0 wt%, 40 °C ST-20, 4.0 wt%, 60 °C

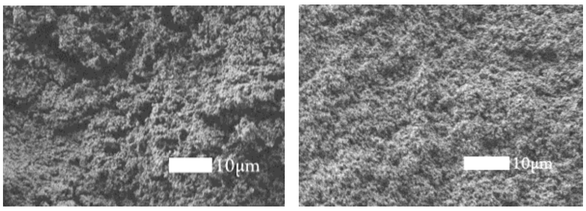

ST-20, 6.0 wt%, 40 °C ST-20, 6.0 wt%, 60 °C

Figure S1. SEM images of 3AZ-ST-20 porous polymer composites (SiO_2 colloids, SiO_2 feed, and preparation temperature), 3AZ monomer concentration: 20 wt%.

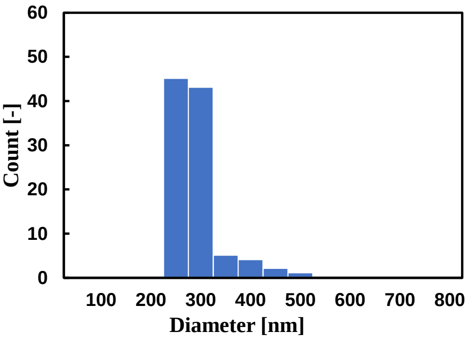

2.0 wt%, 20 °C

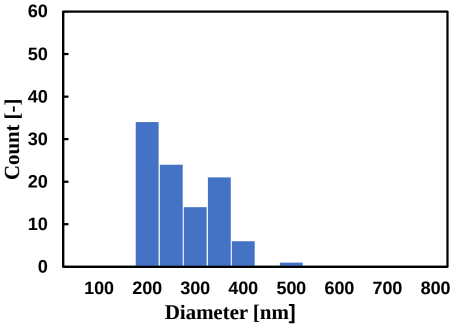

2.0 wt%, 40 °C

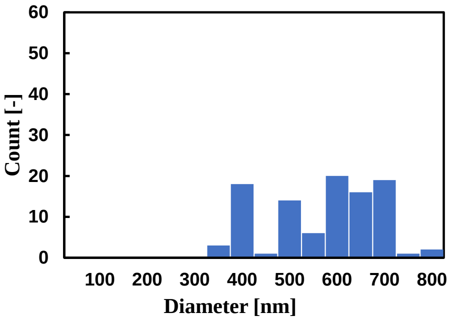

4.0 wt%, 40 °C

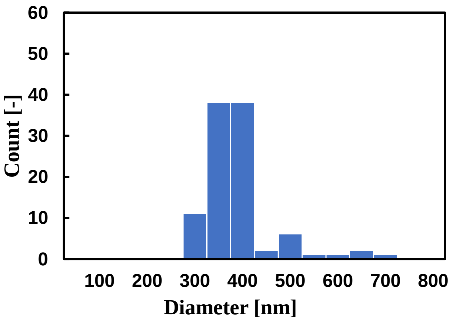

6.0 wt%, 40 °C

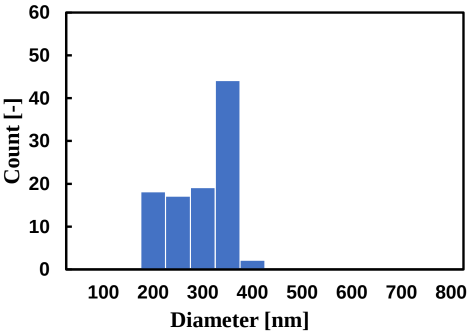

2.0 wt%, 60 °C

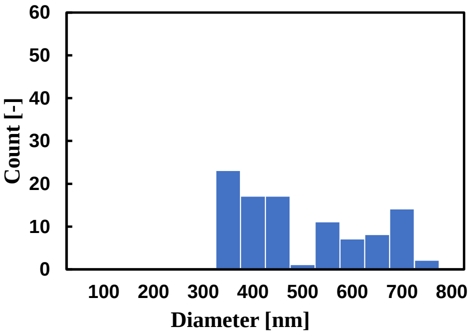

4.0 wt%, 60 °C

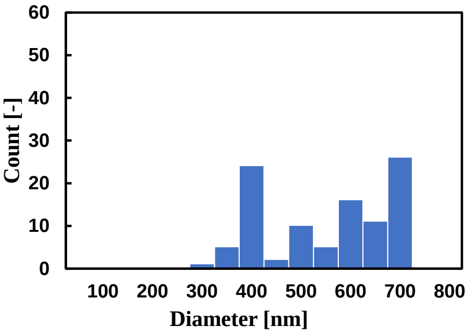

6.0 wt%, 60 °C

Figure S2-1. Histograms of particle diameter of 3AZ-ST-20 porous polymer composites.

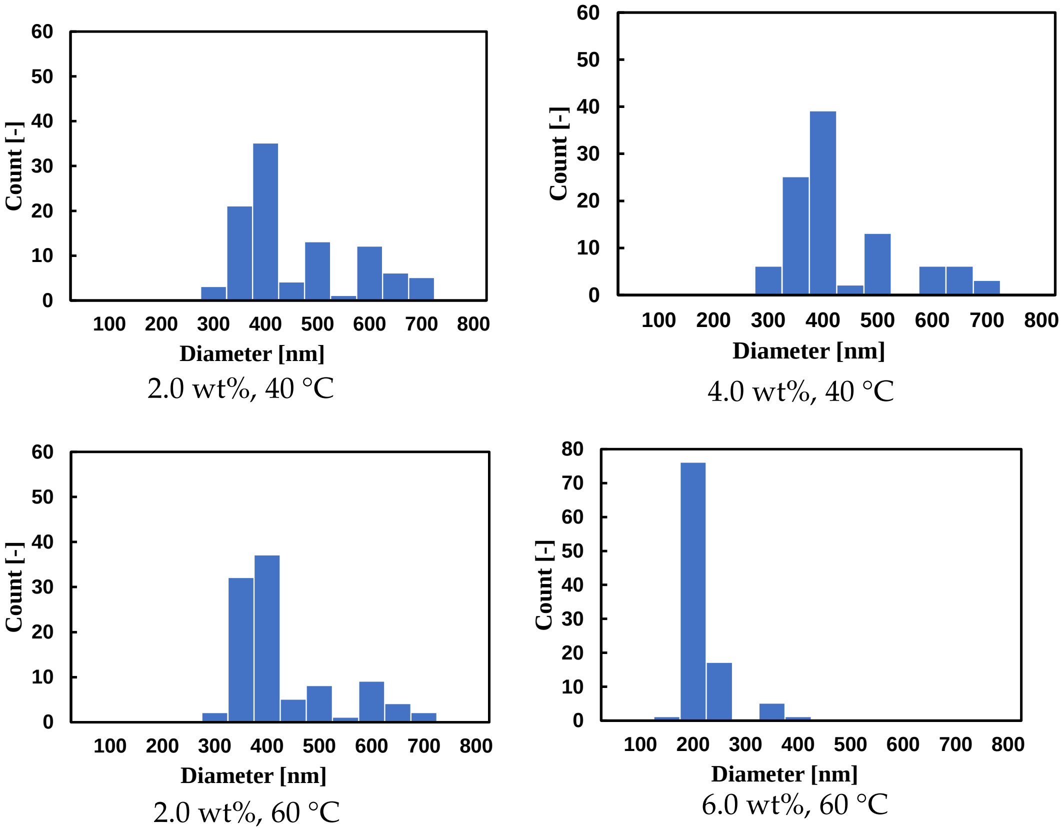

Figure S2-2. Histograms of particle diameter of 3AZ-ST-N porous polymer composites.

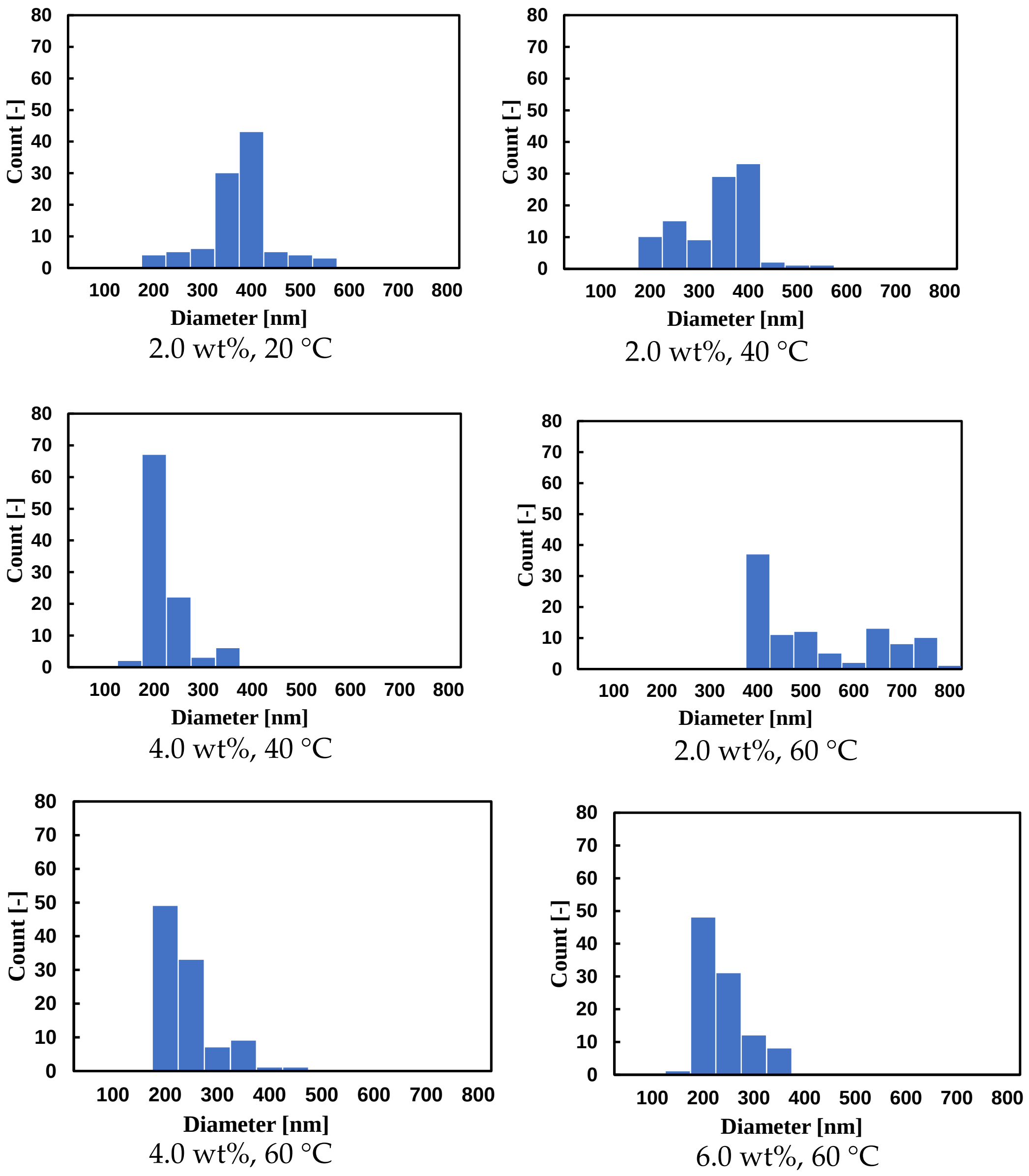

Figure S2-3. Histograms of particle diameter of 3AZ-ST-C porous polymer composites.

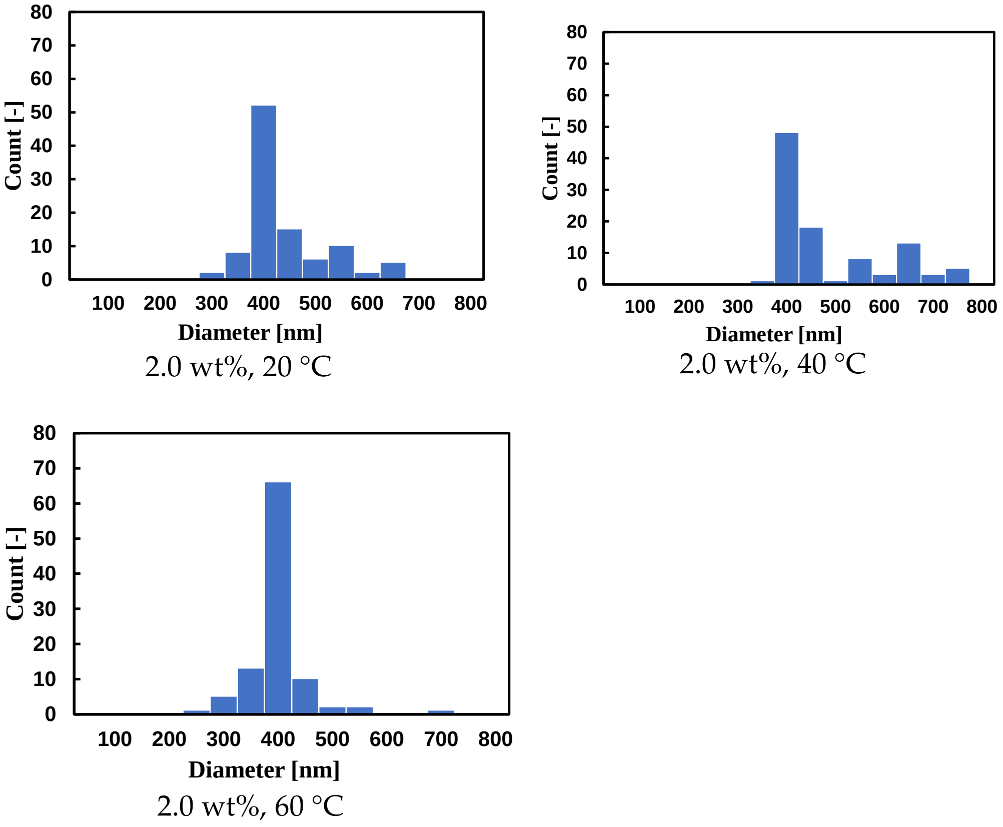

Figure S2-4. Histograms of particle diameter of 3AZ-ST-O porous polymer composites.

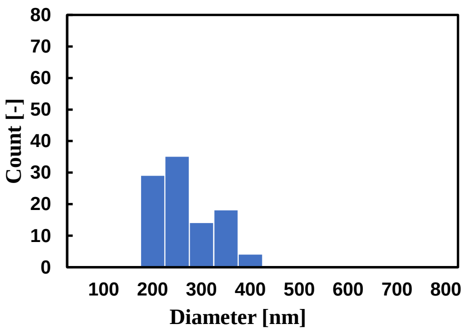

2.0 wt%, 20 °C

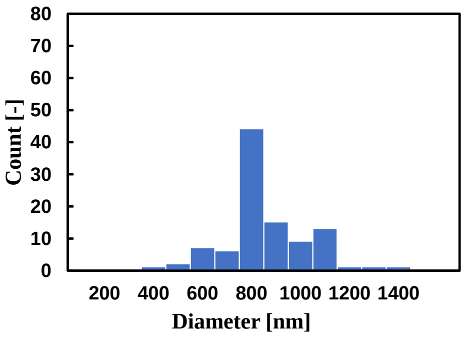

2.0 wt%, 40 °C

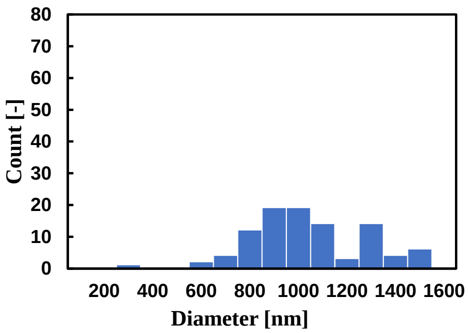

2.0 wt%, 60 °C

Figure S2-5. Histograms of particle diameter of 3AZ-ST-O porous polymer composites.

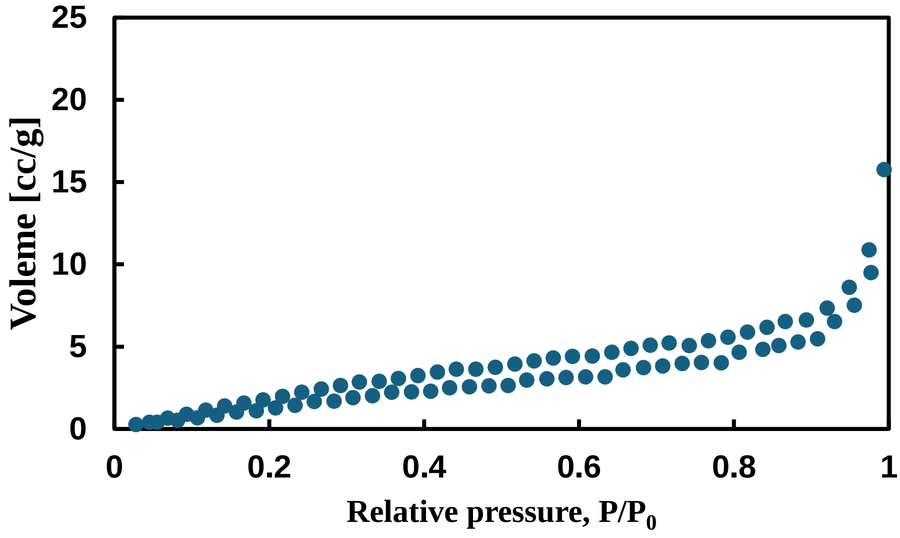

Figure S3. Adsorption/desorption isotherm of 3AZ-ST-20 porous polymer composite (run 8).

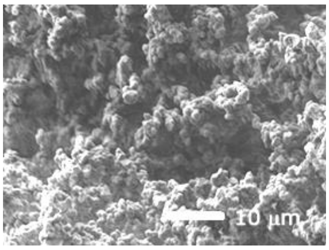

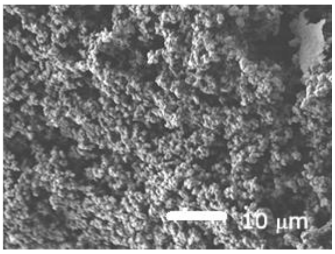

1.0 wt%, 20°C   2.0 wt%, 20°C

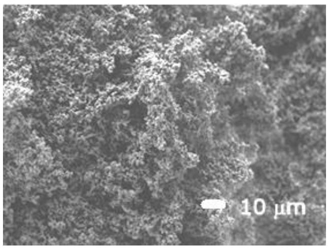

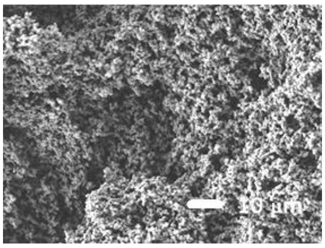

1.0 wt%, 40°C   2.0 wt%, 40°C

Figure S4. SEM images of 3AZ-ZrO_2 porous polymer composites (oZrO_2 feed, preparation temperature), 3AZ monomer concentration: 20 wt%.

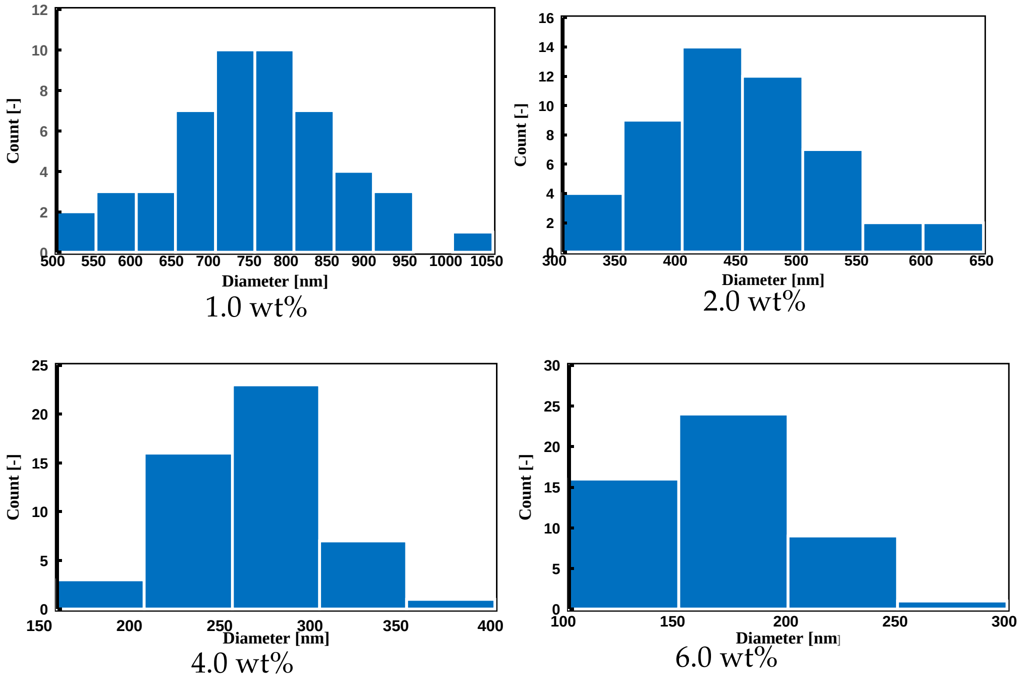

Figure S5-1. Histograms of particle diameter of 3AZ-ZrO_2 porous polymer composites. Prepared at 20 °C

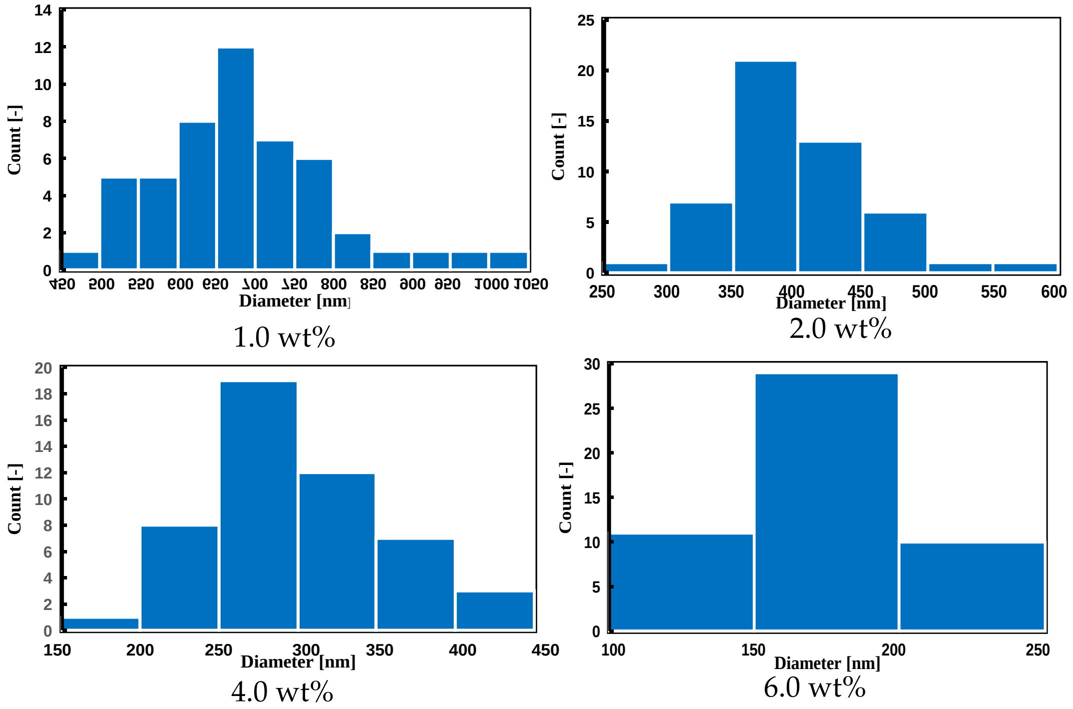

Figure S5-2. Histograms of particle diameter of 3AZ-ZrO_2 porous polymer composites prepared at 40 °C.

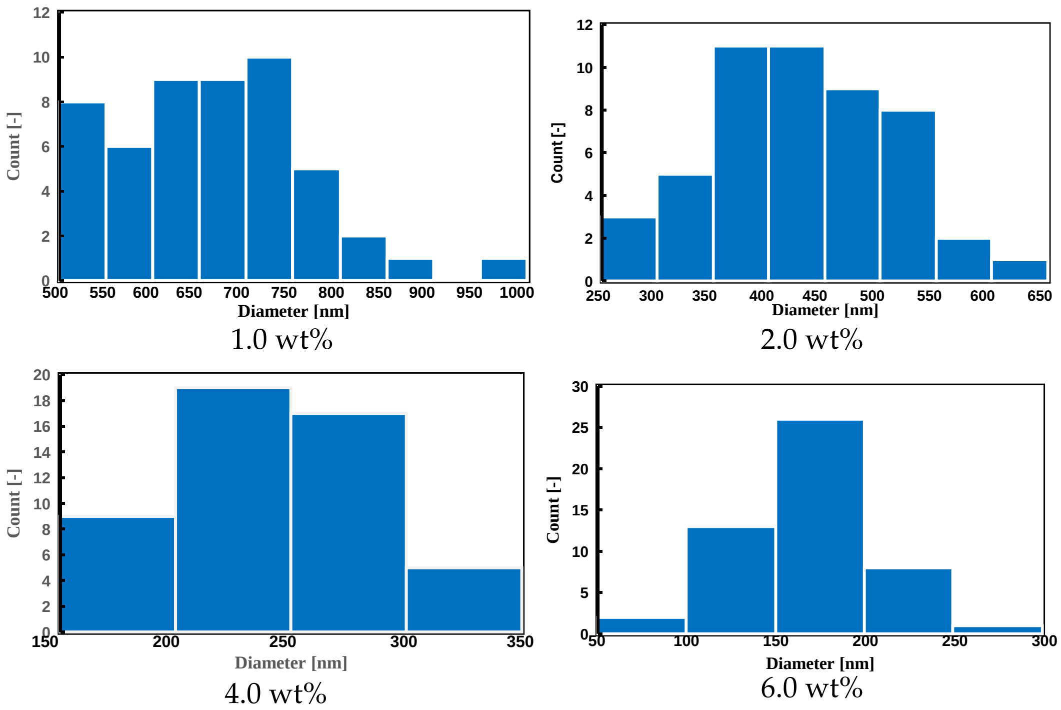

Figure S5-3. Histograms of particle diameter of 3AZ-ZrO_2 porous polymer composites prepared at 60 °C.

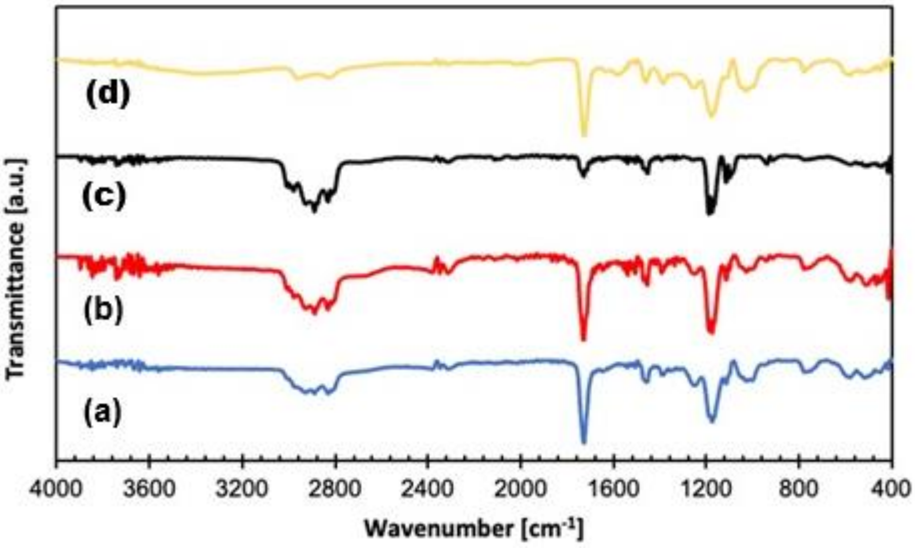

Figure S6. FT-IR spectra of 3AZ-ZrO_2 porous polymer nanocomposites (ZrO_2 nanoparticle feed and preparation temperature); (a) 6.0 wt%, 20 °C, (b) 6.0 wt%, 40 °C, (c) 6.0 wt%, 60 °C, and (d) 2.0 wt%, 60 °C.
